# An accurate prediction model to identify undiagnosed at-risk patients with COPD: a cross-sectional case-finding study

**DOI:** 10.1038/s41533-019-0135-9

**Published:** 2019-05-28

**Authors:** Kang-Cheng Su, Hsin-Kuo Ko, Kun-Ta Chou, Yi-Han Hsiao, Vincent Yi-Fong Su, Diahn-Warng Perng, Yu Ru Kou

**Affiliations:** 1 Institute of Physiology, School of Medicine, National Yang-Ming University, Taipei, Taiwan, ROC; 2 Department of Chest Medicine, Taipei Veterans General Hospital, Taipei, Taiwan, ROC; 3 Center of Sleep Medicine, Taipei Veterans General Hospital, Taipei, Taiwan, ROC; 4 Department of Internal Medicine, Taipei City Hospital Yangming Branch, Taipei, Taiwan, ROC; 5 School of Medicine, National Yang-Ming University, Taipei, Taiwan, ROC

**Keywords:** Chronic obstructive pulmonary disease, Respiratory signs and symptoms, Physical examination

## Abstract

Underuse or unavailability of spirometry is one of the most important factors causing underdiagnosis of COPD. We reported the development of a COPD prediction model to identify at-risk, undiagnosed COPD patients when spirometry was unavailable. This cross-sectional study enrolled subjects aged ≥40 years with respiratory symptoms and a smoking history (≥20 pack-years) in a medical center in two separate periods (development and validation cohorts). All subjects completed COPD assessment test (CAT), peak expiratory flow rate (PEFR) measurement, and confirmatory spirometry. A binary logistic model with calibration (Hosmer-Lemeshow test) and discrimination (area under receiver operating characteristic curve [AUROC]) was implemented. Three hundred and one subjects (development cohort) completed the study, including non-COPD (154, 51.2%) and COPD cases (147; stage I, 27.2%; II, 55.8%; III–IV, 17%). Compared with non-COPD and GOLD I cases, GOLD II-IV patients exhibited significantly higher CAT scores and lower lung function, and were considered clinically significant for COPD. Four independent variables (age, smoking pack-years, CAT score, and percent predicted PEFR) were incorporated developing the prediction model, which estimated the COPD probability (*P*_COPD_). This model demonstrated favorable discrimination (AUROC: 0.866/0.828; 95% CI 0.825–0.906/0.751–0.904) and calibration (Hosmer-Lemeshow *P* = 0.332/0.668) for the development and validation cohorts, respectively. Bootstrap validation with 1000 replicates yielded an AUROC of 0.866 (95% CI 0.821–0.905). A *P*_COPD_ of ≥0.65 identified COPD patients with high specificity (90%) and a large proportion (91.4%) of patients with clinically significant COPD (development cohort). Our prediction model can help physicians effectively identify at-risk, undiagnosed COPD patients for further diagnostic evaluation and timely treatment when spirometry is unavailable.

## Introduction

Chronic obstructive pulmonary disease (COPD) is a key cause of morbidity and mortality worldwide.^1,2^ However, the disease has been considerably underdiagnosed.^3^ The causes of underdiagnosis include low awareness regarding COPD in the general population and among doctors in charge, as well as the low use of spirometry.^4^ The absence of patients in clinics is probably the leading cause because they might lack symptom perception and disease knowledge. In addition, a high proportion of underdiagnosis occurs in primary care settings.^5–7^ Underuse or unavailability of spirometry is the most common cause of underdiagnosis in primary care settings.^4,8,9^ In Taiwan, a recent nationwide telephone interview survey of the general population for COPD prevalence revealed that up to 6.1% might have COPD, but less than 2% had undergone spirometry examination.^10^ Hence, an effective COPD case-finding strategy other than spirometry is urgently required. Moreover, among the identified cases of COPD, symptomatic COPD cases with more severe airflow limitations have been termed as “clinically significant COPD”. Patients with clinically significant COPD may benefit from available treatments.^11,12^ The U.S. National Heart, Lung, and Blood Institute task force suggested that initially identifying these cases may have a greater benefit-to-cost ratio when implementing a case-finding strategy.^12^ Thus, the early identification of COPD and clinically significant COPD are important.

Currently, standardized spirometry measurements in primary care settings are usually hindered due to the complex, time-consuming procedures and high expenses required. By contrast, the measurement of peak expiratory flow rate (PEFR) by using a handheld flow meter is simple and cheap. Previous studies have reported that the PEFR can identify COPD cases in terms of an area under the receiver operating characteristic curve (AUROC) of approximately 0.66–0.88.^13–15^ This wide AUROC range indicates that the PEFR should be cautiously used for identifying COPD. PEFR reduction arbitrarily indicates lung function impairment. However, such reduction is not exclusive to obstructive lung disease, but is a common and important clue for airway obstruction, particularly among those with exposure risk and respiratory symptoms. Moreover, COPD case findings are only recommended in symptomatic subjects.^16^ Thus the application of questionnaires may potentially compensate for this drawback. A questionnaire can provide information regarding exposure risk and respiratory symptoms. Existing validated questionnaires can identify undiagnosed COPD cases with a corresponding AUROC of approximately 0.71–0.82.^17–19^ The COPD assessment test (CAT) is a short and guideline-recommended questionnaire in the management of COPD patients.^16,20–22^ The CAT evaluates the severity of respiratory symptoms as well as the impact on the quality of life. Thus, the CAT might potentially serve as a case-finding tool. Both the PEFR and CAT are common tools in real-life practice. Combining the PEFR and CAT may provide a new and precise tool for identifying COPD cases. This possibility is worthy of further investigation.

Previous studies have applied two-stage approaches, using various screening questionnaires to select high-risk cases and then, conducted PEFR measurement with these cases. These studies concluded that the aforementioned strategy improved the accuracy of COPD identification.^13,15,23^ However, the strategy may potentially miss COPD cases in groups categorized as low-risk by the questionnaires, who might be unaware of the disease or be less perceptive to its symptoms. Thus, we initiated a one-step COPD case-finding study by inviting all at-risk subjects to complete the CAT, PEFR measurement, and confirmatory spirometry. We aimed to develop a logit model by using easily assessed variables, including the age, smoking status, PEFR, and CAT score, to estimate the probability of COPD (*P*_COPD_) and clinically significant COPD. Moreover, the robustness of the final model was examined through sensitivity analysis.

## Results

### Patient characteristics

In the development cohort, 373 consecutive subjects were invited and 301 completed the study (Supplementary Fig. 1a). Most of the development cohort subjects (242, 80.4%) directly came from the community without any referrals, and the others were referred from non-pulmonary clinics (39, 13%) at our hospital and from general practitioners (GPs) (20, 6.6%) in the community. The subjects were categorized into the non-COPD (154, 51.2%) and newly diagnosed COPD (147, 48.8%) groups (Table 1). Of the 147 COPD cases, 40 (27.2%) were categorized as stage I (post-bronchodilation [BD] forced expiratory volume in first second (FEV_1_) ≥80%), 82 (55.8%) were stage II (50% ≤ post-BD FEV_1_ <80%), and 25 (17%) were stage III–IV (post-BD FEV_1_ <50%) as per the severity classification proposed by the Global Initiative for Chronic Obstructive Lung Disease (GOLD).^24^ Compared with the non-COPD subjects and COPD GOLD stage I patients, GOLD stages II–IV COPD patients had significantly higher symptoms (CAT score) and lower lung function variables (including the PEFR, pre-BD, and post-BD FEV_1_, and forced vital capacity [FVC]). By contrast, GOLD stage I patients were similar to non-COPD subjects in terms of the symptoms and lung function (Table 1). Thus, GOLD stages II–IV COPD patients were considered clinically significant for COPD. For all the subjects, the CAT score was weakly and negatively associated with the percent predicted PEFR (%PEFR) and post-BD percent predicted FEV_1_ (%FEV_1_) (Pearson’s *r* = −0.379 and −0.409, respectively; both *P* *<* 0.001). However, the post-BD %FEV_1_ was strongly correlated with the %PEFR (Pearson’s *r* = 0.739, *P* < 0.001). For the validation cohort, 142 subjects (from the community [98, 69%], intra-hospital referrals via non-pulmonary clinics [26, 18.3%], and GP referrals [18, 12.7%]) of the 171 invited completed the study (Supplementary Fig. 1b). This cohort included the non-COPD (95, 66.9%) and COPD (47, 33.1%) groups (Supplementary Table 1). The characteristics of the subjects in this cohort were similar to those in the development cohort, and clinically significant COPD was also represented by the GOLD II–IV COPD patients.

**Table 1:** Characteristics of the study subjects categorized by spirometry-confirmed COPD in the development cohort

	All	Total subjects			COPD patients divided by GOLD stage			
		COPD	Non-COPD	*P* ^a^	GOLD I	GOLD II	GOLD III–IV	*P* ^b^
Numbers	301	147	154		40	82	25	
Age, years	70.7 ± 13.2	75.2 ± 11.3	66.5 ± 13.5	<0.001	77.9 ± 9.9^c^	75.0 ± 11.2^c^	72.3 ± 10.8	<0.001
Gender, male (%)	287 (95)	139 (95)	148 (96)	0.524^c^	39 (98)	77 (94)	23 (92)	0.652^c^
Current smoker (%)	128 (43)	56 (38)	72 (47)	0.129^c^	14 (35)	30 (37)	12 (48)	0.315^c^
Smoking pack-years	45.4 ± 25.0	50.6 ± 26.0	40.3 ± 20.1	<0.001	52.3 ± 29.9^d^	49.5 ± 25.9^d^	51.7 ± 19.8	0.004
*Peak flow meter*								
Best PEFR (L/min)	383 ± 148	290 ± 120	472 ± 113	<0.001	403 ± 101^d^	270 ± 85^d,e^	174 ± 96^d,e,f^	<0.001
PEFR, % pred.	79 ± 28	63 ± 25	95 ± 20	<0.001	89 ± 19	57 ± 18^d,e^	38 ± 16^d,e,f^	<0.001
*AT score*								
Total	8.1 ± 6.9	10.2 ±8.0	6.1 ± 4.9	<0.001	5.7 ± 4.1	11.4 ± 8.4^d,e^	13.2 ± 8.6^d,e^	<0.001
Cough	1.8 ± 1.4	2.0 ± 1.4	1.6 ± 1.3	0.011	1.7 ± 1.2	2.2 ± 1.6^d^	1.9 ± 1.3	0.018
Phlegm	1.8 ± 1.4	2.0 ± 1.5	1.5 ± 1.3	0.001	1.6 ± 1.2	2.1 ± 1.5^d^	2.4 ± 1.6^d^	0.001
Chest tightness	1.0 ± 1.3	1.2 ± 1.5	0.8 ± 1.0	0.002	0.7 ± 1.1	1.4 ± 1.6^d,e^	1.4 ± 1.6	<0.001
Breathlessness	1.1 ± 1.5	1.6 ± 1.7	0.7 ± 1.0	<0.001	0.3 ± 0.6	1.9 ± 1.8^d,e^	2.4 ± 1.6^d,e^	<0.001
Activity limitation	0.6 ± 1.2	0.9 ± 1.5	0.3 ± 0.9	<0.001	0.3 ± 0.9	0.9 ± 1.6^d^	1.5 ± 1.6^d,e^	<0.001
Confidence	0.3 ± 1.0	0.5 ± 1.2	0.6 ± 1.1	<0.001	0.1 ± 0.2	0.6 ± 1.4^d,e^	1.0 ± 1.5^d,e^	<0.001
Sleep	0.7 ± 1.2	0.8 ± 1.3	0.6 ± 1.1	0.114	0.4 ± 0.8	1.0 ± 1.4^e^	1.1 ± 1.2	0.009
Energy	0.9 ± 1.2	1.2 ± 1.4	0.6 ± 1.0	<0.001	0.6 ± 1.0	1.4 ± 1.5^d,e^	1.4 ± 1.3^d,e^	<0.001
*Spirometry, pre-BD*								
FEV_1_ (L)	1.96 ± 0.75	1.47 ± 0.53	2.44 ± 0.62	<0.001	1.93 ± 0.50^d^	1.43 ± 0.39^d,e^	0.87 ± 0.20^d,e,f^	<0.001
FEV_1_, % pred.	81 ± 24	66 ± 20	96 ± 16	<0.001	90 ± 12^d^	62 ± 9^d,e^	39 ± 9^d,e,f^	<0.001
FVC (L)	2.83 ± 0.80	2.52 ± 0.77	3.12 ± 0.72	<0.001	2.99 ± 0.74	2.47 ± 0.68^d,e^	1.95 ± 0.66^d,e,f^	<0.001
FVC, % pred.	84 ± 17	79 ± 19	89 ± 14	<0.001	95 ± 14	76 ± 15^d,e^	62 ± 17^d,e,f^	<0.001
FEV_1_/FVC (%)	68 ± 14	58 ± 12	78 ± 7	<0.001	65 ± 7^d^	58 ± 11^d,e^	47 ± 14^d,e,f^	<0.001
*Spirometry, post-BD*								
FEV_1_ (L)	2.05 ± 0.75	1.56 ± 0.55	2.52 ± 0.61	<0.001	2.05 ± 0.52^d^	1.50 ± 0.38^d,e^	0.94 ± 0.22^d,e,f^	<0.001
FEV_1_, % pred.	85 ± 23	70 ± 20	99 ± 16	<0.001	96 ± 12	66 ± 8^d,e^	42 ± 8^d,e,f^	<0.001
FVC (L)	3.02 ± 0.93	2.82 ± 1.07	3.20 ± 0.72	<0.001	3.22 ± 0.73	2.68 ± 0.68^d,e^	2.21 ± 0.66^d,e,f^	<0.001
FVC, % pred.	89 ± 17	86 ± 19	92 ± 15	0.003	103 ± 14^d^	83 ± 16^d,f^	70 ± 17^d,e,f^	<0.001
FEV_1_/FVC (%)	68 ± 15	56 ± 12	79 ± 6	<0.001	63 ± 5^d^	56 ± 11^d,e^	44 ± 14^d,e,f^	<0.001

Data are presented as means ± standard deviation

*% pred*. percent predicted value, *BD* bronchodilation, *CAT* COPD assessment test, *COPD* chronic obstructive pulmonary disease, *FEV*_*1*_ forced expiratory volume in the first second, *FVC* forced expiratory capacity, *PEFR* peak expiratory flow rate

^a^Independent *t*-test, COPD vs. non-COPD

^b^One-way ANOVA test, compare 4 groups: non-COPD, GOLD I, GOLD II, and GOLD III-IV

^c^Chi-square test

^d^Post-hoc Bonferroni test, *P* < 0.05, vs. non-COPD

^e^Post-hoc Bonferroni test, *P* < 0.05, vs. GOLD I

^f^Post-hoc Bonferroni test, *P* < 0.05, vs. GOLD II

### Model development and sensitivity analysis

**Fig. 1:**
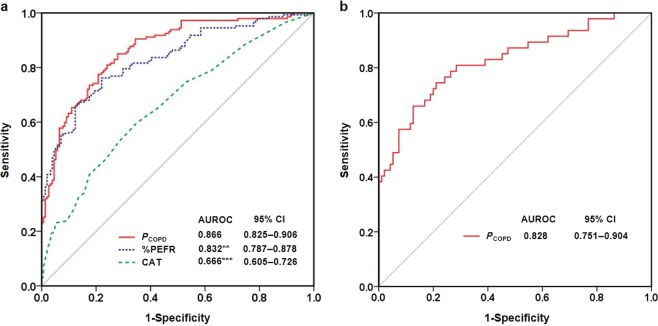
Diagnostic accuracy according to the ROC curve analysis. The ROC curve and AUROC value of the selected diagnostic modality in the development (**a**) and validation (**b**) cohorts. **P < 0.01, ***P < 0.001, vs. *P*_COPD_. Statistical evaluations were performed using MedCalc based on the methodology from DeLong et al. ROC, receiver operating characteristic curve; AUROC, area under the ROC; CI, conference interval; %PEFR, percent predicted peak expiratory flow rate; CAT, COPD assessment test; *P*_COPD_, probability of COPD

In the present study, there was no missing data in those who completed the study in both cohorts. The factors considered for the diagnosis of COPD are listed in Table 2. Each factor was entered into the univariate logistic regression, which revealed that the age, smoking pack-years, best PEFR, %PEFR, total and individual CAT scores (except sleep) were significant variables for COPD. Among these variables, collinearity existed between the best PEFR and %PEFR (Pearson’s *r* = 0.892, *P* < 0.001) as well as between the total CAT score and individual CAT score (Pearson’s *r* = 0.625-0.737, all *P* < 0.001). We adopted the age, smoking pack-years, %PEFR, and CAT for multivariate logistic regression, and all four of these variables were statistically significant and remained in the model (Table 2). Thus, these four variables were incorporated into a logit model, which could estimate the *P*_COPD_ (Table 3). The results of sensitivity analysis indicated that the four-variable combined model (*P*_COPD_) reached the highest diagnostic accuracy of COPD in terms of the AUROC (0.866). The removal of any variables from the model decreased its accuracy (Supplementary Fig. 2). To simplify the model, a single variable was used to predict COPD according to the %PEFR or CAT, which resulted in AUROC values of 0.832 and 0.666, respectively. However, both the AUROC values were significantly inferior to the value of *P*_COPD_ (Fig. 1a). The cut-offs and corresponding predictive performance of the *P*_COPD_, %PEFR, and CAT are presented in Table 4.

**Table 2:** Variables associated with the diagnosis of COPD in the development cohort

	Univariate				Multivariate			
	*β*	Odds ratio	95% CI	*P* ^a^	*β*	Odds ratio	95% CI	*P* ^a^
Age, years	0.055	1.06	1.04–1.08	<0.001	0.045	1.05	1.02–1.07	<0.001
Sex, male	− 0.35	0.71	0.24–2.08	0.526				
Current smoker	− 0.355	0.7	0.44–1.11	0.129				
Smoking pack-years	0.017	1.02	1.01–1.03	0.001	0.015	1.02	1.00–1.03	0.016
Best PEFR (L/min)	− 0.012	0.99	0.98–0.99	<0.001				
Predicted PEFR (%)	− 0.056	0.95	0.93–0.96	<0.001	−0.049	0.95	0.94–0.97	<0.001
*CAT score*								
Total	0.103	1.11	1.06–1.16	<0.001	0.056	1.06	1.00–1.12	0.037
Cough	0.213	1.24	1.05–1.46	0.012				
Phlegm	0.282	1.33	1.12–1.57	0.001				
Chest tightness	0.289	1.34	1.11–1.61	0.003				
Breathlessness	0.469	1.6	1.33–1.92	<0.001				
Activity limitation	0.447	1.56	1.24–1.97	<0.001				
Confidence	0.506	1.66	1.21–2.28	0.002				
Sleep	0.154	1.17	0.96–1.41	0.116				
Energy	0.416	1.52	1.23–1.86	<0.001				

*β* regression coefficient, *CAT* COPD assessment test, *CI* confidence interval, *COPD* chronic obstructive pulmonary disease, *PEFR* peak expiratory flow rate

^a^Wald test in Binary logistic regression

**Table 3:** Estimating the probability of COPD in the development cohort

Data source used in this model^a^	Independent variables				Estimated *P*_COPD_	COPD yes/no	Post-BD FEV1/FVC	Pre-BD %FEV1
	Age	Pack-years	CAT	%PEFR				
*From means of our cohort*								
Non-COPD subjects	67	40	6	95	0.23			
COPD subjects	75	51	10	63	0.75			
*From selected study subjects*								
Subject A	71	53	3	79	0.45	Yes	0.56	63
Subject B	67	20	4	74	0.36	No	0.71	82
Subject C	49	86	13	63	0.65	Yes	0.62	59
Subject D	47	21	2	78	0.14	No	0.75	79

*%PEFR* percent predicted peak expiratory flow rate, *CAT* COPD assessment test, *COPD* chronic obstructive pulmonary disease, *P*_COPD_ probability of COPD

^a^Entering the values of the four variables into a preset computer program immediately calculates the probability of COPD

**Table 4:** Performance of different modalities to identify undiagnosed COPD in the development cohort

	Sensitivity	Specificity	PPV	NPV
*Identification of COPD*				
CAT ≥ 7^a^	60	65	62	63
%PEFR < 79%^a^	76	78	77	77
*P*_COPD_ ≥ 0.40	82	73	75	81
0.44^a^	78	79	78	79
0.50	72	83	80	76
0.60	67	86	83	74
0.65	63	90	86	72
0.70	61	91	87	71

*%PEFR* percent predicted peak expiratory flow rate, *CAT* COPD assessment test, *COPD* chronic obstructive pulmonary disease, *NPV* negative predictive value, *P*_*COPD*_ probability of COPD, *PPV* positive predictive value

^a^Indicates the best cutoff value determined by Youden index

### Equation and performance of the COPD prediction model

By using the aforementioned four independent variables, the logit model to determine *P*_COPD_ was expressed as follows:

1 $${\text{logit}}\left( {{{P}}_{{\text{COPD}}}} \right) = {\it{f}}\left( {{x}} \right) = - {0}.51 + \left( {{0}{.}{045} \times {\text{age}}} \right) + \left( {{0}{.}{015} \times {\text{pack}} - {\text{years}}} \right)+\left( {{0}{.056} \times {\text{CAT}}} \right)+\left( {-{ 0}{.}{049} \times \% {\text{PEFR}}} \right)$$

The aforementioned equation was transformed as follows:

2 $$\begin{array}{l}{{P}}_{{\mathrm{COPD}}} = \exp \left[ \begin{array}{l} - 0.51 + \left( {0.045 \times {\mathrm{age}}} \right)\,{\mathrm{ + }}\,\left( {0.015 \times {\mathrm{pack}} - {\mathrm{years}}} \right)\\ +\, \left( {0.056\, \times {\mathrm{CAT}}} \right)\,{\mathrm{ + }}\,\left( { - 0.049 \times \% {\mathrm{PEFR}}} \right)\end{array} \right]\\ {\mathrm{/}}\left\{ {1{\mathrm{ + }}\exp \left[ \begin{array}{l} - 0.51 + \left( {0.045\, \times {\mathrm{age}}} \right)\,{\mathrm{ + }}\,\left( {0.015 \times {\mathrm{pack}} - {\mathrm{years}}} \right)\\ \left( {0.056\, \times {\mathrm{CAT}}} \right)\,{\mathrm{ + }}\,\left( { - 0.049 \times \% {\mathrm{PEFR}}} \right)\end{array} \right]} \right\}\end{array}$$

The estimated *P*_COPD_ can be readily calculated by entering the four variables into preset computer software. The goodness-of-fit Hosmer-Lemeshow test was non-significant for both the development and validation cohorts (*P* = 0.332 and 0.668, respectively), which indicated accurate calibration. The discrimination was also favorable in terms of the AUROC for both the development (0.866) and validation (0.828) cohorts (Fig. 1). Bootstrap validation revealed similar discrimination (AUROC: 0.865, 95% conference interval [CI] 0.821–0.905).

### Predictive performance and cutoffs

**Fig. 2:**
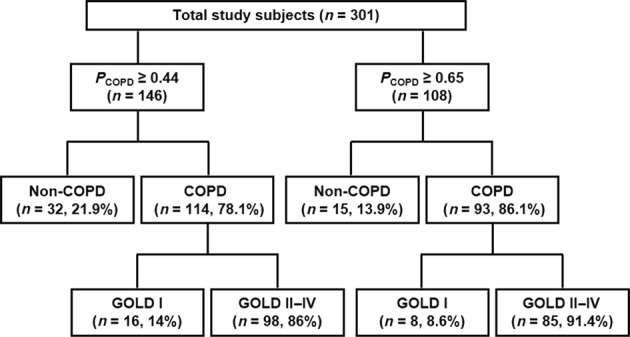
Distributions of study subjects categorized by the potential probability of COPD. COPD, chronic obstructive pulmonary disease; GOLD, Global Initiative for Chronic Obstructive Lung Disease; *P*_COPD_, probability of COPD

The mean estimated *P*_COPD_ was 0.75 and 0.23 for the COPD patients and non-COPD subjects, respectively (Table 3). *P*_COPD_ ≥0.44 exhibited favorable diagnostic accuracy for identifying cases with COPD. The cut-off of *P*_COPD_ ≥0.44 correctly identified 77.6% of total COPD cases, with the missing COPD cases (false negatives; those with *P*_COPD_ <0.44 but actually had COPD) having a few symptoms (mean CAT score 6.3) and preserved lung function (mean post-BD FEV_1_ 90%; Supplementary Table 2). In comparison to *P*_COPD_ ≥0.44, *P*_COPD_ ≥ 0.65 identified COPD patients with higher specificity (90 vs. 79%; Table 4), a lower false-positive rate (13.9 vs. 21.9%; Fig. 2) and a higher proportion of clinically significant COPD patients (GOLD II-IV, 91.4 vs. 86.0%; Fig. 2). A cut-off CAT score of <7 and <10 resulted in 40.1 and 59.2% missing COPD cases, respectively, with a mean post-BD %FEV_1_ of 79 and 77%, respectively (Supplementary Table 2).

## Discussion

Four variables were employed in this study, namely the age, smoking pack-years, CAT, and %PEFR to form an accurate prediction model for identifying undiagnosed COPD. The favorable model performance indicates that the prediction model is robust and accurate. When using only a single variable to identify COPD, the CAT is inadequate. A higher accuracy was obtained when using the %PEFR alone than when using the CAT alone. However, the four-variable model demonstrated the highest accuracy, offering a one-step, rapid estimation of *P*_COPD_. Moreover, with a tight cut-off, the prediction model could identify clinically significant COPD with a high degree of specificity. Therefore, the prediction model can serve as a clinically practical strategy for identifying cases of COPD.

The prevalence of COPD among older adults is high, and the diagnosis of COPD is based on exposure risks, respiratory symptoms, and airflow limitations according to the guideline.^16^ Previous epidemiological studies have reported that increasing age and smoking pack-years are strongly associated with COPD.^25^ Similarly, case-finding studies have found that COPD cases have significantly higher CAT scores^26^ and lower PEFRs^23,27^ than non-COPD cases. To our knowledge, this study is unique that the aforementioned four variables have been employed as a one-step approach to identify COPD in at-risk subjects. *P*_COPD_ can be calculated immediately by entering the four variables into an equation by using a software program. The satisfactory accuracy and simplicity of our model offer the potential for wide application. The model could help physicians identify patients at risks of COPD, particularly in primary care settings where confirmatory spirometry is unavailable.

Confirmation of airflow limitation by spirometry is required for COPD diagnosis.^16^ After considering the benefits of improving patient outcomes and altering the disease process, the US Preventive Services Task Force did not recommend screening for COPD in asymptomatic subjects through questionnaires and spirometry.^28^ Moreover, the identification of patients with clinically significant COPD is likely to have considerable beneficial treatment effects.^11,12^ In this study, GOLD stages II–IV patients were considered as clinically significant for COPD because they had higher symptom scores and lower lung function than non-COPD subjects and COPD GOLD stage I patients. Early identification and management may be an appropriate strategy for this patient population. In our model, a *P*_COPD_ cut-off of ≥0.44 correctly identified a substantial proportion of COPD cases, with the missing cases (false negatives) exhibiting a few symptoms and preserved lung function (Supplementary Table 2). In comparison to *P*_COPD_ ≥ 0.44, *P*_COPD_ ≥ 0.65 identified COPD patients with a higher specificity and proportion of clinically significant patients. The missing COPD cases for *P*_COPD_ ≥ 0.65 were early stage COPD and less symptomatic patients, who may require alternative interventions rather than urgent medication. Considering cost-effectiveness, *P*_COPD_ ≥ 0.65 may be valuable for identifying subjects at risk of COPD, who may require further diagnostic evaluation and timely treatment in primary care settings where spirometry is unavailable.

Currently, PEFR reduction was one of the most common alternative tools suggestive of the presence of airflow limitation and employed in COPD case-finding studies.^13–15,23,27,29^ The pre-BD %PEFR was highly correlated with the pre- and post-BD %FEV_1_ in this study and previous reports.^13,15,30^ We observed that the PEFR alone identified undiagnosed COPD with a reasonable predictive performance, which was very close to the predictive performance reported by Tian (our data vs. Tian: AUROC 0.832 vs. 0.879).^14^ However, the predictive performance with the PEFR is variable, with AUROC ranging from 0.66 to 0.88.^13–15^ Different clinical settings, standard instructions for how to use a peak flow meter, and various devices may influence the accurate measurement of the PEFR and diagnostic accuracy.

The CAT score could enable discrimination between non-COPD and COPD cases (Table 1 and Supplementary Table 1), which is consistent with the previous results reported by Raghavan et al.^26^ The correlation between the CAT score and the lung function variables (%PEFR or %FEV1) was weak, and the predictive performance of CAT were not satisfactorily observed in this study, with similar results reported in previous studies.^31,32^ A CAT score of ≥7 yielded an optimal cut-off for the diagnostic accuracy in this study. The GOLD strategy considers that COPD cases with CAT scores ≥10 are symptomatic.^16^ However, both a CAT score <7 or <10 resulted in a high proportion of missing COPD cases with compromised lung function (Supplementary Table 2). Thus, the CAT alone is inadequate as a screening tool for identifying undiagnosed CPOPD cases.

It may be argued that this study was conducted in a medical center, where, the patient population may differ from those in primary care settings. However, outpatients in medical center in Taiwan are atypical of those in other countries, which is ascribed to the unique healthcare system in Taiwan. This government-run, single-payer health insurance system is characterized by compulsory coverage for all citizens, convenient accessibility, and low costs with almost all medically necessary services covered. The system has a weak gatekeeper role and no restrictive referral regulations. Thus, outpatients have freedom to choose any specialist in any hospital, including a medical center, without a referral.^33^ This loose regulation results in most Taiwanese people visiting a doctor directly at a medical center. The Taiwan National Health Insurance Administration announced that a substantially high proportion of outpatients in medical centers sought specialist care without any referrals. In this study, those without referrals and with GP-referrals accounted for over 80% of the subjects in both cohorts. Although not completely identical, our study population was similar to the population in primary care settings. Thus, the predictive model has the potential to be applied in the community.

Our study has certain strengths. The study design was based on a one-step approach to identify undiagnosed COPD patients and the availability of assessment tools for future use in primary care settings. Moreover, the *P*_COPD_ can be quickly measured through computer software. This study also has certain limitations. First, we lacked information regarding subjects’ underlying comorbidities, which may have affected the CAT score, PEFR, and *P*_COPD_. Second, further evaluation of the prediction model is required in genuine primary care settings to expand the model generalizability. Third, some of our COPD subgroups (GOLD I and GOLD III–IV) had limited patient numbers, and the CAT might have varied over time. Thus, these results should be individualized and cautiously applied for initiating COPD treatment. Finally, whether subjects with high *P*_COPD_ but without spirometric confirmation should start treatment requires further investigation.

In conclusion, we developed and validated an accurate COPD prediction model using the age, smoking-pack years, %PEFR, and CAT score. The model can accurately and rapidly estimate the *P*_COPD_ in at-risk subjects or undiagnosed COPD patients who may require further diagnostic evaluation and timely treatment when spirometry is unavailable. The developed prediction model may be a cost-effective tool for use in COPD case-finding strategies.

## Methods

### Study design

This cross-sectional, observational study was conducted at a medical center, namely Taipei Veterans General Hospital, Taiwan, from November 2011 to March 2014 for the development cohort and from December 2017 to December 2018 for the validation cohort. The study subjects were invited in pulmonary outpatient clinics, where their demographic information, chest X-rays, CAT questionnaires (Chinese version^34^), PEFR measurements, and diagnostic spirometry (Supplementary Fig. 1 for the study flow) were obtained. All the participants completed the study flow on the same day. This study was approved by the Institutional Review Board of Taipei Veterans General Hospital (ID: 2011-07-010IC for the development cohort and 2017-07-006C for the validation cohort). As the course of this study was part of our routine clinical service, the requirement for patient informed consent was waived in the development cohort. Subsequently, for a more rigorous study, informed consent was obtained in the validation cohort.

### Study subjects

Eligible subjects were aged ≥ 40 years, had a history of smoking ≥ 20 pack-years, presented with chronic respiratory symptoms (at least one of cough, phlegm, or dyspnea), and denied a previous history of chronic respiratory illness (including COPD, asthma, bronchiectasis, lung cancer, lung fibrosis, pulmonary tuberculosis, and any neuromuscular or spinal disease that affected lung function). Subjects were excluded if they had an acute respiratory infection 2 weeks prior to enrollment, exhibited significant abnormality on chest radiographs, or were unable/unwilling to undergo peak flow meter testing and/or spirometry. Finally, the study subjects were categorized into non-COPD and COPD with distinct GOLD obstructive stages for pairwise comparisons of different variables.

### Measurements of lung function

The PEFR measurement was performed using a Mini-Wright peak flow meter (Micropeak, Micro Medical Limited, Rochester, UK) according to the ERS recommendations.^35^ The best PEFR was adopted from three correct blows when patients exerted maximal expiratory efforts in a standing position. Following at least a 1-h break, the patients completed confirmatory spirometry for the diagnosis of COPD. Pre-BD and post-BD (20–30 min after inhalation of 400 μg of salbutamol via a Ventolin metered dose inhaler with a spacer; GlaxoSmithKline, Brentford, UK) spirometry (Spiro Medics system 2130; SensorMedics; Anaheim, CA, USA) was performed in accordance with the guideline from the American Thoracic Society/European Respiratory Society.^36^ The diagnosis of COPD was confirmed by a post-BD ratio (FEV_1_ over FVC FVC) <0.7.^16,24^

### Sample size estimation

The best practice for sample size estimation in the development cohort is to have at least 10 outcome events per variable estimated^37–39^ (i.e., the ratio of COPD patients to the selected variables is 10). We selected 12 variables, including the age, sex, smoking intensity, PEFR, and eight symptoms in the CAT, which corresponded to a target number of COPD cases of 120. According to the review data in our pulmonary clinics, approximately 40% of patients who met the inclusion criteria were diagnosed as GOLD-defined COPD,^16,24^ irrespective of severity classification. Therefore, the estimated sample size in the development cohort had to reach a minimum value of 300. However, the required sample size in validation cohorts is not well understood.^37^ We calculated the required size according to the AUROC value. For a type I error of 0.05 and a power of 0.9, we assumed to reach an AUROC value of 0.7. The required minimal sample size was therefore at least 116 (estimated using MedCalc software, see Supplementary Fig. 3).

### Statistical analysis

Data are presented as means ± SD or a number (%), as appropriate. Continuous variables were compared using a *t*-test or one-way analysis of variance, followed by a Bonferroni test for pairwise comparisons. Categorical data were evaluated using a chi-square test. The association between two continuous variables was evaluated through Pearson’s correlation. A binary logistic regression model using the enter method was applied to examine the independent variables related to the diagnosis of COPD and to generate an equation for estimating the *P*_COPD_. Therefore, the accuracy of using different modalities to diagnose COPD could be determined through ROC curve analysis. The optimal cut-off of the selected modality was calculated using the Youden index to determine the sensitivity, specificity, positive predictive value, and negative predictive value.

Subsequently, the logit model to estimate the *P*_COPD_ was employed using the independent variables for the highest accuracy. Thus, the log odds ratio of subjects with or without COPD is expressed as follows:

3 $${\mathrm{ln}}\left[{P}_{\mathrm{COPD}}/\left(1 - P_{\mathrm{COPD}}\right)\right] = {\mathrm{logit}}\left({P}_{\mathrm{COPD}}\right) = f(x) = \beta_{0} + \beta_{1} {\mathrm{X}}_{1} + \beta_{2}{\mathrm{X}}_{2} + \ldots+ \beta_{i}{\mathrm{X}}_{i},$$

where *β*_0_ is the coefficient of the constant and *β*_*i*_ is the coefficient(s) of the independent variable(s) *X*_*i*_. This equation can be transformed as follows:

4 $${{P}}_{{\mathrm{COPD}}} = e^{f\left( {\mathrm{X}} \right)}{\mathrm{/}}\left( {1 + {\mathrm{e}}^{f\left( {\mathrm{X}} \right)}} \right),$$

where *P*_COPD_ can be directly calculated.^40^ We applied sensitivity analysis to investigate the influence of dropping different variables from the prediction model (*P*_COPD_) on the diagnostic accuracy represented by the AUROC in the development cohort. We also examined the prediction model by using the AUROC for discrimination, Hosmer-Lemeshow goodness-of-fit test for calibration, and resampling bootstrap validation with 1000 replicates. Statistical analysis was performed using SPSS for Windows, version 20.0 (IBM Corp., Armonk, NY, USA). The comparison of the AUROC values (based on the methodology from DeLong et al.^41^) and sample size estimation according to the AUROC value were performed using MedCalc version 17.5.5 (MedCalc Software bvba, Ostend, Belgium). The AUROC of the resampling bootstrap was estimated using R statistical software (version 3.5.1, R Foundation for Statistical Computing, Vienna, Austria). A two-sided *P* *<* 0.05 was considered significant.

## Supplementary information

Supplementary Material

Dataset

## Data Availability

All data generated or analyzed during this study are included in this published article (and its supplementary information files).

## Supplementary Information

**Supplementary information** accompanies the paper on the *npj Primary Care Respiratory Medicine* website (10.1038/s41533-019-0135-9).
