# Supplementary material for: An accurate prediction model to identify undiagnosed at-risk patients with COPD: a cross-sectional case-finding study
Source: NPJ Prim Care Respir Med. 2019 May 28;29:22. doi: 10.1038/s41533-019-0135-9 (PMC6538645; doi:10.1038/s41533-019-0135-9)
Supplement: Supplementary file 1 — Supplementary Material [file 41533_2019_135_MOESM1_ESM.pdf]

SUPPLEMENTARY INFORMATION

An accurate prediction model to identify undiagnosed at-risk patients with COPD: a cross-sectional case-finding study

Supplementary Figure 1 Study flow in the development (a) and validation (b) cohorts. An abnormal CXR includes overt bronchiectasis (n= 11 and 4), interstitial lung disease (n= 8 and 3), pleural effusion (n= 6 and 2), lobar infiltration (n= 4 and 2), lung mass (n= 3 and 1), or mediastinal lesions (n= 2 and 0) in the development and validation cohorts, respectively. *CXR* chest X-ray, *CAT* COPD assessment test, *COPD* chronic obstructive pulmonary disease

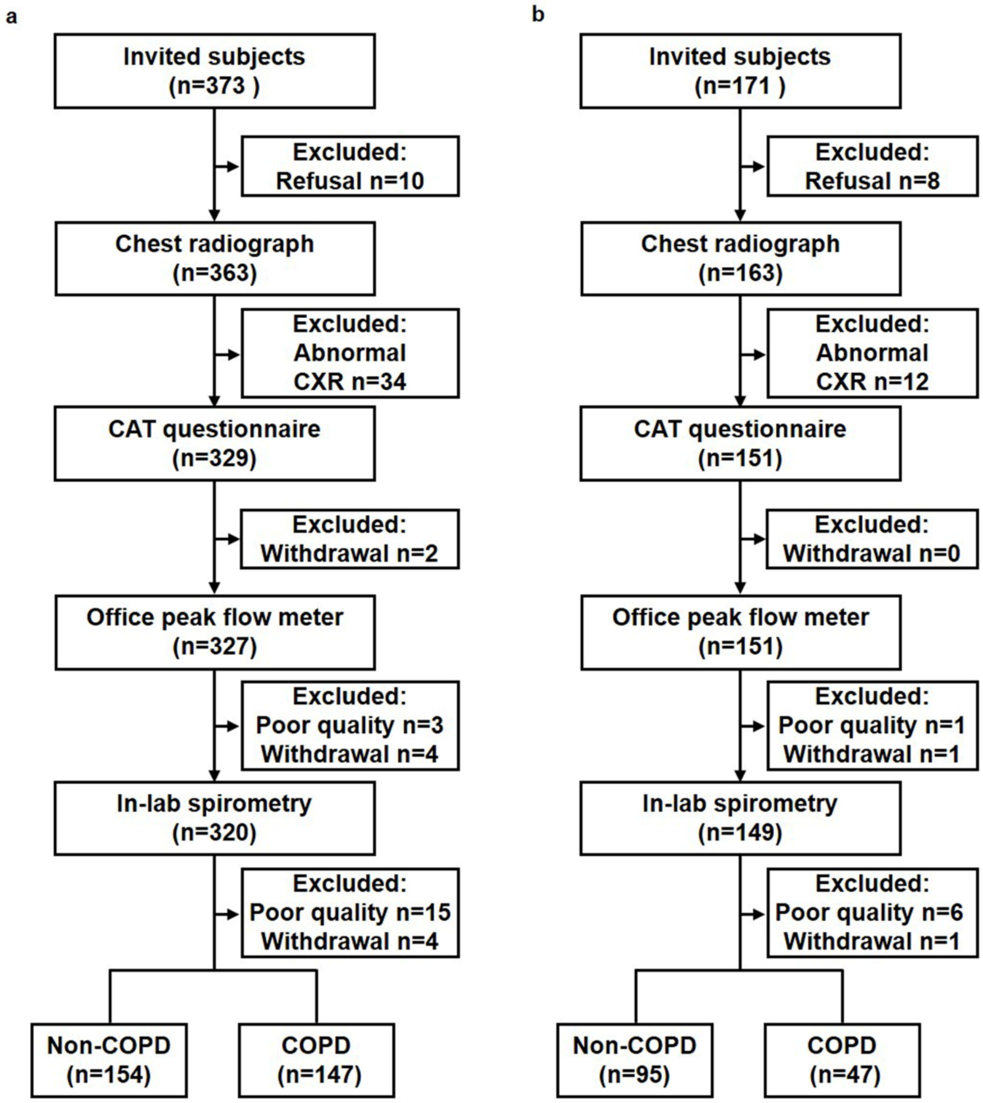

Supplementary Figure 2 Sensitivity analysis for the influence of dropping different variables from the prediction model (P_{COPD}) on the diagnostic accuracy of COPD represented by the area under the receiver operating characteristic curve (AUROC) in the development cohort. P_{COPD} probability of COPD, *CI* conference interval

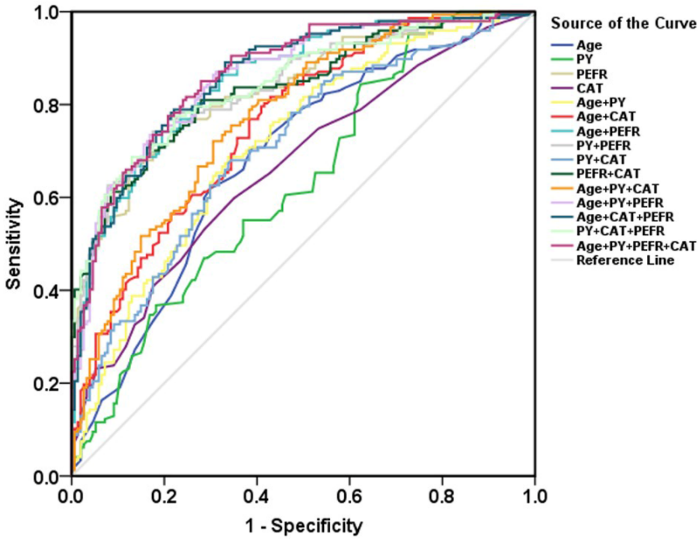

Tested Result variable(s)	AUROC	95% CI
Single variable		
Age	0.684	0.624 - 0.744
PY	0.631	0.569 - 0.694
PEFR	0.832	0.787 - 0.878
CAT	0.666	0.605 - 0.726
Two variables		
Age+PY	0.715	0.658 - 0.773
Age+CAT	0.756	0.702 - 0.809
Age+PEFR	0.853	0.811 - 0.895
PY+PEFR	0.842	0.798 - 0.886
PY+CAT	0.707	0.649 - 0.765
PEFR+CAT	0.834	0.789 - 0.88
Three ariables		
Age+PY+CAT	0.772	0.72 - 0.824
Age+PY+PEFR	0.860	0.819 - 0.901
Age+CAT+PEFR	0.860	0.819 - 0.901
PY+CAT+PEFR	0.845	0.801 - 0.889
Four variables (P_{COPD})		
Age+PY+PEFR+CAT	0.866	0.825 - 0.906

Supplementary Figure 3 Sample size estimation for the validation cohort. Given a type I error of 0.05, a type II error of 0.1 (equal to a power of 0.9), the null hypothesis value of 0.5, the ratio of subjects with versus without COPD of 3, the required minimal sample size is at least 116 (calculated using the MedCalc software).

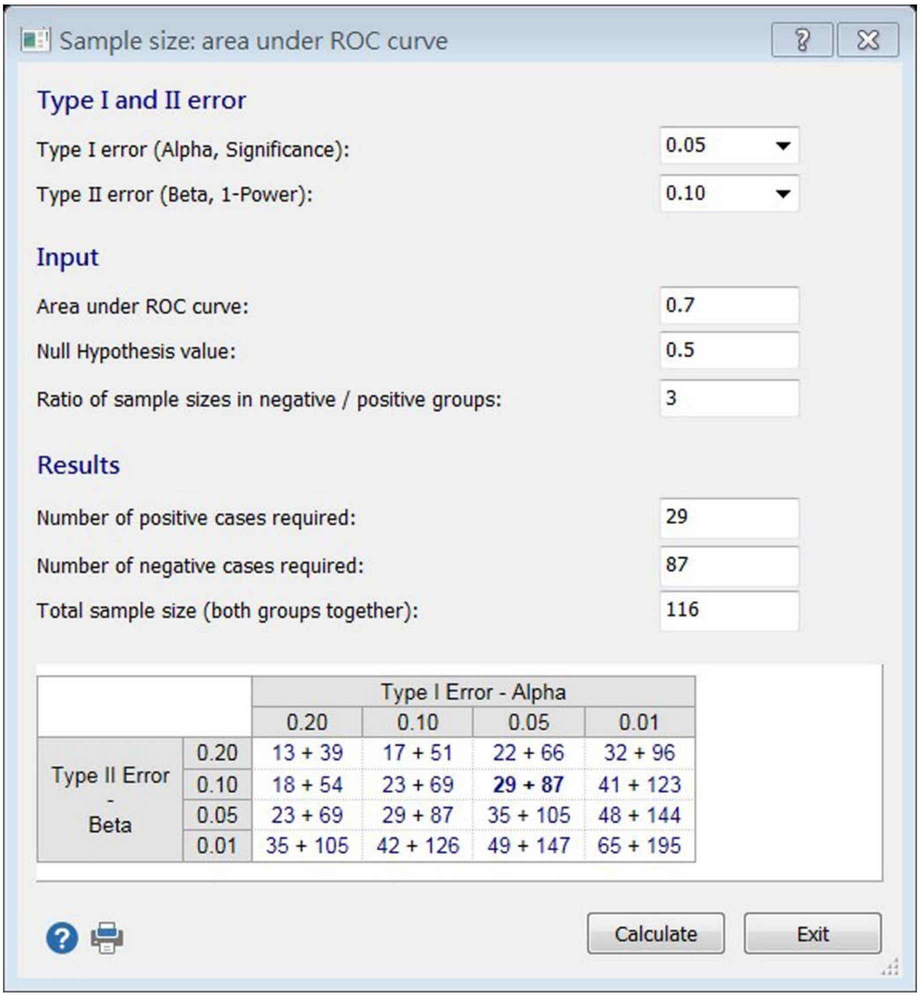
Sample size: area under ROC curve

Type I and II error

Type I error (Alpha, Significance): 0.05

Type II error (Beta, 1-Power): 0.10

Input

Area under ROC curve: 0.7

Null Hypothesis value: 0.5

Ratio of sample sizes in negative / positive groups: 3

Results

Number of positive cases required: 29

Number of negative cases required: 87

Total sample size (both groups together): 116

		Type I Error - Alpha			
		0.20	0.10	0.05	0.01
Type II Error - Beta	0.20	13 + 39	17 + 51	22 + 66	32 + 96
	0.10	18 + 54	23 + 69	**29 + 87**	41 + 123
	0.05	23 + 69	29 + 87	35 + 105	48 + 144
	0.01	35 + 105	42 + 126	49 + 147	65 + 195

Calculate Exit

Supplementary Table 1. Characteristics of the study subjects categorized by spirometry-confirmed COPD in the validation cohort

		Total subjects			COPD patients divided by GOLD stage			
	All	COPD	Non-COPD	*P*[a]	GOLD I	GOLD II	GOLD III-IV	*P*[b]
Numbers	142	47	95		26	18	3	
Age, years	65.2±11.4	71.1±11.7	62.3±10.1	<0.001	73.5±11.7[d]	68.3±11.7	67±9.8	<0.001
Gender, male (%)	140 (99)	47 (100)	93 (98)	0.446[c]	26 (100)	18 (100)	3 (100)	0.800[c]
Current smoker (%)	53 (37)	17 (36)	36 (38)	0.842[c]	9 (35)	8 (44)	0 (0)	0.518[c]
Smoking pack-years	43.1±20.3	46.1±20.2	41.6±20.3	0.215	43.0±17.4	44.2±19.1	84.5±9.3[d,e,f]	0.004
Peak flow meter								
Best PEFR, L/min	466±128	366±134	516±92	<0.001	384±144[d]	358±118[d]	253±101[d]	<0.001
PEFR, % pred.	92±22	75±25	100±14	<0.001	80±25[d]	72±23[d]	52±18[d]	<0.001
CAT score								
Total	5.0±4.2	6.4±4.6	4.3±3.8	0.004	4.4±3.5	8.3±4.4[d,e]	12.7±5.5[d,e]	<0.001
Cough	1.4±1.2	1.5±1.2	1.3±1.2	0.239	1.0±1.1	2.3±1.1[d,e]	1.7±0.6	0.002
Phlegm	1.3±1.1	1.6±1.1	1.2±1.1	0.015	1.4±1.2	1.9±1.0[d]	1.7±0.6	0.033
Chest tightness	0.7±0.9	0.9±1	0.5±0.9	0.040	0.6±0.8	1.0±0.9	2.3±1.5[d,e]	0.002
Breathlessness	0.8±1.1	1.3±1.3	0.5±0.9	< 0.001	0.7±1.0	1.8±1.2[d,e]	3.3±0.6[d,e]	<0.001
Activity limitation	0.1±0.4	0.3±0.6	0.1±0.3	0.002	0.2±0.5	0.2±0.5	1.3±0.6[d,e,f]	<0.001
Confidence	0.1±0.2	0.1±0.3	0.1±0.2	0.126	0.1±0.2	0.1±0.2	0.7±0.6[d,e,f]	<0.001
Sleep	0.3±0.8	0.2±0.7	0.4±0.8	0.336	0.1±0.3	0.4±1	0.3±0.6	0.443
Energy	0.5±0.9	0.5±0.9	0.4±0.9	0.428	0.4±0.8	0.6±0.8[d]	1.3±1.5[d]	0.298
Spirometry, pre-BD								
FEV_1, liter	2.56±0.78	1.95±0.67	2.86±0.64	<0.001	2.23±0.65[d]	1.71±0.47[d,e]	0.95±0.37[d,e]	<0.001
FEV_1, % pred.	96±20	80±21	103±15	<0.001	95±11[d]	66±11[d,e]	39±12[d,e,f]	<0.001
FVC, liter	3.32±0.90	2.92±0.88	3.52±0.84	<0.001	3.23±0.92	2.63±0.61[d]	1.90±0.59[d]	<0.001
FVC, % pred.	91±17	85±19	95±16	0.001	96±15	73±11[d,e]	57±15[d,e]	< 0.001
FEV_1/FVC (%)	77±10	66±8	82±6	<0.001	69±4[d]	65±8[d]	50±16[d,e,f]	<0.001
Spirometry, post-BD								
FEV_1, liter	3.42±9.8	2.02±0.67	2.91±0.64	<0.001	2.30±0.67[d]	1.80±0.44[d]	0.98±0.31[d,e]	<0.001
FEV_1, % pred.	77±10	66±8	82±6	<0.001	69±4	65±8[d,e]	50±16[d,e,f]	<0.001
FVC, liter	3.41±0.86	3.08±0.87	3.57±0.81	0.001	3.39±0.94	2.80±0.54[d]	2.01±0.47[d,e]	<0.001
FVC, % pred.	94±16	90±19	96±15	0.030	101±15	78±10[d,e]	61±12[d,e]	<0.001
FEV_1/FVC (%)	76±10	65±7	82±5	<0.001	68±3[d]	63±7[d]	50±16[d,e,f]	<0.001

Data are presented as means ± standard deviation

% pred. percent predicted value, *BD* bronchodilation, *CAT* COPD assessment test, *COPD* chronic obstructive pulmonary disease, *FEV_1* forced expiratory volume in the first second, *FVC* forced expiratory capacity, *PEFR* peak expiratory flow rate

[a]Independent t-test, COPD *vs.* non-COPD

[b]One-way ANOVA test, compare 4 groups: non-COPD, GOLD I, GOLD II, and GOLD III-IV

[c]Chi-square test

[d]Post-hoc Bonferroni test, $P < 0.05$, *vs.* non-COPD

[e]Post-hoc Bonferroni test, $P < 0.05$, *vs.* GOLD I

[f]Post-hoc Bonferroni test, $P < 0.05$, *vs.* GOLD II

Supplementary Table 2. Characteristics of misdiagnosed (false-negative) cases of COPD categorized by different modalities in the development cohort

	COPD cases with			
	$P_{COPD} < 0.44$	PEFR ≥ 79%	CAT < 7	CAT < 10
Numbers (% of total COPD)	33 (22.4)	35 (23.8)	59 (40.1)	87 (59.2)
Age, years	72.5 ± 112.2	75.9 ± 9.4	76.7± 9.3	75.8 ± 10.3
Smoking pack-years	44.2 ± 18.5	49.4 ± 9.4	51.9 ± 28.5	51.8 ± 27.7
Peak flow meter				
Best PEFR, L/min	443.3 ± 75.6	442.8 ± 72.6	340.5 ± 120.4	328.4 ± 112.3
PEFR, % pred.	97.0 ± 14.0	98.0 ± 12.0	72.5 ± 26.8	70.2 ± 25.0
Total CAT scores	6.3 ± 5.5	6.4 ± 5.4	3.6 ± 1.6	5.0 ± 2.5
Spirometry, pre-				
FEV_1, liter	2.00 ± 0.49	1.99 ± 0.47	1.64 ± 0.53	1.63 ± 0.51
FEV_1, % pred.	85.9 ± 15.2	88.7 ± 15.0	74.6 ± 20.3	72.8 ± 19.7
FVC, liter	3.01 ± 0.78	2.98 ± 0.74	2.74 ± 0.70	2.74 ± 0.73
FVC, % pred.	90.8 ± 17.8	92.1 ± 18.0	85.9 ± 17.4	84.9 ± 17.3
FEV_1/FVC (%)	66.5 ± 5.3	66.7 ± 5.7	59.8 ± 11.1	59.4 ± 11.4
Spirometry, post-BD				
FEV_1, liter	2.10 ± 0.54	2.08 ± 0.51	1.73 ± 0.55	1.71 ± 0.53
FEV_1, % pred.	90.2 ± 17.7	93.2 ± 16.5	79.0 ± 21.1	76.9 ± 20.6
FVC, liter	3.21 ± 0.82	3.19 ± 0.74	2.95 ± 0.71	2.93 ± 0.74
FVC, % pred.	97.0 ± 18.9	98.8 ± 18.3	92.4 ± 18.1	90.8 ± 18.1
FEV_1/FVC (%)	65.1 ± 3.3	65.1 ± 3.9	58.6 ± 9.7	58.4 ± 10.0

Data are presented as means ± standard deviation

% pred. percent predicted value, *BD* bronchodilation, *CAT* COPD assessment test, *COPD* chronic obstructive pulmonary disease, *FEV_1* forced expiratory volume in the first second, *FVC* forced expiratory capacity, *PEFR* peak expiratory flow rate
